# Metabolomic profiling reveals deep chemical divergence between two morphotypes of the zoanthid *Parazoanthus axinellae*

**DOI:** 10.1038/srep08282

**Published:** 2015-02-06

**Authors:** Nadja Cachet, Grégory Genta-Jouve, Julijana Ivanisevic, Pierre Chevaldonné, Frédéric Sinniger, Gérald Culioli, Thierry Pérez, Olivier P. Thomas

**Affiliations:** 1 Institut de Chimie de Nice - EEIC, UMR 7272 CNRS, Université de Nice-Sophia Antipolis, Parc Valrose, 06108 Nice, France; 2 Laboratoire de Pharmacognosie et de Chimie des Substances Naturelles, UMR CNRS 8638 COMETE, Université Paris Descartes, 4 Avenue de l'Observatoire 75006 Paris, France; 3 Institut Méditerranéen de Biodiversité et d'Ecologie Marine et Continentale, UMR 7263 CNRS, IRD, Aix Marseille Université, Avignon Université, Station Marine d'Endoume, Rue Batterie des Lions, 13007 Marseille, France; 4 Japan Agency for Marine-Earth Science and Technology, 224-3 Aza-Toyohara, Nago City, Okinawa 905-2172, Japan; 5 Tropical Biosphere Reseach Center, University of the Ryukyus, 3422 Sesoko, Motobu, Okinawa 905-0227, Japan; 6 MAPIEM, EA 4323 Université de Toulon, 83957 La Garde, France

## Abstract

Metabolomics has recently proven its usefulness as complementary tool to traditional morphological and genetic analyses for the classification of marine invertebrates. Among the metabolite-rich cnidarian order Zoantharia, *Parazoanthus* is a polyphyletic genus whose systematics and phylogeny remain controversial. Within this genus, one of the most studied species, *Parazoanthus axinellae* is prominent in rocky shallow waters of the Mediterranean Sea and the NE Atlantic Ocean. Although different morphotypes can easily be distinguished, only one species is recognized to date. Here, a metabolomic profiling approach has been used to assess the chemical diversity of two main Mediterranean morphotypes, the “slender” and “stocky” forms of *P. axinellae*. Targeted profiling of their major secondary metabolites revealed a significant chemical divergence between the morphotypes. While zoanthoxanthin alkaloids and ecdysteroids are abundant in both morphs, the “slender” morphotype is characterized by the presence of additional and bioactive 3,5-disubstituted hydantoin derivatives named parazoanthines. The absence of these specific compounds in the “stocky” morphotype was confirmed by spatial and temporal monitoring over an annual cycle. Moreover, specimens of the “slender” morphotype are also the only ones found as epibionts of several sponge species, particularly *Cymbaxinella damicornis* thus suggesting a putative ecological link.

Marine natural products chemistry is a dynamic field of research which has experienced explosive growth in the last decades and is continuing to evolve. However, the biological and ecological functions of marine secondary metabolites are still poorly understood. Being the result of long evolutionary processes of biosynthetic pathway refinement, secondary metabolites are considered as products of natural selection and their diversity has been tentatively used in chemotaxonomy, complementary to morphological characters and/or genetic markers in systematics. For instance, in basal metazoans such as Porifera, systematics can be very challenging. Therefore, an increasing number of integrative taxonomical works on Porifera now successfully consider biochemical datasets in parallel to molecular or morphological ones^1^,^2^,^3^,^4^,^5^.

The main objective of chemotaxonomy is to propose potential synapomorphic chemical markers at different taxonomic ranks. This is sometimes antagonistic to the main objectives of natural products research which focuses on the description of new compounds with potential applications, especially in the pharmaceutical industry. Therefore, in the past, mainly novel and bioactive molecules have been reported, omitting the description of the high chemical diversity in metabolomic fingerprints of marine invertebrate extracts and missing putative synapomorphic chemical characters. Metabolomic fingerprinting has proved to be efficient to detect a large diversity of compounds and to provide a comprehensive and rapid assessment of the chemical composition of a given biological sample (cells, tissue, organ, whole organism)^6^,^7^. This approach has been recently used in phytochemistry and microbiology to classify plants or prokaryotic strains^8^, while it has rarely been applied to discriminate between marine metazoan species. To the best of our knowledge, the only applications of such a holistic chemotaxonomical approach have been conducted on sponges in order to highlight subtle chemical divergence between sister species^4^,^9^,^10^, to discriminate among cryptic species^1^, and even to support molecular phylogenetic hypotheses^5^. However, since metabolomics allows high-throughput, large dynamic range and highly specific analyses of complex biological extracts, the use of such an approach is likely to expand across a number of different taxonomic groups.

Although sponges are the paramount source of marine bioactive metabolites, cnidarians, and especially anthozoans, display high biological and chemical diversity and as such they have been the focus of many promising researches on natural products^11^,^12^. Among them, relatively little is known about zoanthids (Cnidaria, Hexacorallia, Zoantharia) despite the fact that they are common in most shallow and deep marine environments. Within this group, the Mediterranean and North Eastern Atlantic species *Parazoanthus axinellae* (Schmidt, 1862) is among the best studied species. This zoanthid grows in encrusting colonies of soft polyps lacking a skeleton. It is a common organism in sublittoral rocky communities, especially in habitats with low light irradiance, on shaded vertical cliffs, overhangs and at cave entrances. *Parazoanthus axinellae* is a species rather variable in morphology and colour, though with few diagnostic characters^13^. This variability and taxonomic uncertainty has led to the description of five subspecies, none of which being reliably identified^14^. One morphotype, described as *P. axinellae brevi-tentaculatus* Abel, 1959 (subsequently named *P. axinellae brevitentacularis*) can be easily distinguished^14^. Its main features include shorter polyp trunk, short and thick tentacles, together with a more pronounced orange colour. Hereafter, this morphotype is called “stocky” (Figure 1B). Other forms that display a more elongated trunk, longer and thinner tentacles, as well as a lighter colour, will be referred to as “slender” morphotype (Figure 1A). Both morphotypes can occur in sympatry and syntopy on rocky walls, although only the slender form can also be found growing on *Cymbaxinella damicornis*, a demosponge from which the species name derives. DNA taxonomy is now frequently used in situations where morphological examination is inconclusive or confused. However, anthozoans are well known for their slow rate of molecular evolution^15^,^16^. This high conservatism of DNA sequence has direct implications in the identification of species using DNA taxonomy and it was suggested that the molecular markers currently used cannot distinguish between closely related species^17^,^18^,^19^. However, no DNA study has yet surveyed the identity or differences between the different forms of *P. axinellae*.

The secondary metabolome of *P. axinellae* was first studied in the 1970s with the isolation and structure elucidation of polyaromatic alkaloids named zoanthoxanthins and parazoanthoxanthins^20^,^21^,^22^. Recently, a second original family of alkaloids, named parazoanthines, was recovered from the same species^23^. This unexpected finding, after decades of research on the chemistry of *P. axinellae*, led to wonder whether the two morphotypes of *P. axinellae* (“stocky” and “slender”) may be represented by two disparate, specific metabolomic phenotypes. Indeed, it is likely that previous chemical studies have focused on only one of the two morphotypes. In order to confirm or reject this assumption, we studied herein the variability in the secondary metabolome of both *P. axinellae* morphotypes, over time (different seasons) and space (across different geographical regions). In addition, DNA sequence variability among morphs was also investigated.

## Results

### DNA sequences of stocky and slender morphotypes of *Parazoanthus axinellae*

Mitochondrial 16S and 12S rRNA and COI gene sequences obtained from both morphotypes did not show any differences among morphs along the 790 bp, 699 bp and 633 bp sequenced respectively. Similarly the 830 bp of the nuclear ITS-2 were also identical. In addition to these traditionally used markers, the non-coding region within the COI intron (652 bp) and the 3′ part of COI (610 bp), also failed to distinguish between the two morphotypes. Moreover, when compared to a “slender form” of *P. axinellae* from the Atlantic (Ireland), no differences were observed either along 1180 bp of 16S or 627 bp of COI and only 1 bp difference was observed in ITS2 (830 bp) with the Atlantic specimen having a 9 Adenine repeat compared to 8 in both Mediterranean forms.

### Specialized metabolome of *P. axinellae*

The largest panel of secondary metabolites observed in LC-UV-(+)ESIMS profiles for both *P. axinellae* morphotypes was acquired in the retention time window between 10 and 30 min. The three families of *P. axinellae* metabolites were identified according to their UV absorbance and MS fragmentation patterns. Structural characterization of the major metabolites in the defined RT window was performed thus allowing their quantification over an annual cycle in both morphotypes of *P. axinellae.*

The sixteen metabolites **1**-**16** were purified and identified from both morphotypes of *P. axinellae*. Their structure elucidation was based on combined NMR and MS data, and further comparison with literature data. Thus, the three main chemical families are: ecdysteroids including 20-hydroxyecdysone (**1**)^24^, 20-hydroxyecdysone 2-acetate (**2**), 20-hydroxyecdysone 3-acetate (**3**)^25^, and viticosterone E (**4**)^26^; zoanthoxanthins including parazoanthoxanthins C (**5**) and D (**6**), norpseudoparazoanthoxanthin (**7**)^20^,^21^,^22^ and paragracine (**8**)^27^; and parazoanthines (**9-16**) (Figure 2)^23^,^28^.

Two ecdysteroids, 20-hydroxyecdysone (**1**) and gerardiasterone^29^ are present in *S. savaglia* crude extract while zoanthoxanthins and parazoanthines are not found in this species.

### Variation in space of metabolomic fingerprints

Thirteen sites of the Western Mediterranean were sampled for the geographical variation study (Figure 3) and 17 samples of *P. axinellae* “slender” and 9 of *P. axinellae* “stocky” were analyzed using a metabolomic approach.

HPLC-(+)ESIMS metabolomic fingerprints of *P. axinellae* provide evidence for clear chemical divergence between two categories of samples, sorted according to the two morphotypes of *P. axinellae* and not geographical location (Figure 4). This grouping is mainly explained by the detection of parazoanthines (**9-16**) only present in the metabolomic profiles of *P. axinellae* “slender” (Figure 5). The hierarchical classification of *P. axinellae* LC-MS profiles highlighted also a clear divergence between *S. savaglia* and *P. axinellae*, with two well-defined clades within the latter: the “slender” and “stocky” morphotypes (Supporting Information). Whatever the considered geographic location, the variability within morphotype and within chemotype was always found to be lower than the variability observed between them, the “stocky” morphotype exhibiting a much lower inter individual variability.

### Variation in time of targeted secondary metabolites

We compared the concentrations of the three families of compounds all along the year for both morphotypes in the same location near Monaco (Figure 3). A total of 63 specimens were thus analyzed using the same metabolomic approach. For the “stocky” morphotype, the relative proportion of ecdysteroids was the lowest in March, August and September where the proportion of zoanthoxanthins was the highest (Figure 6). Remarkably, parazoanthines **9-16** were absent throughout the year from the metabolomic windows of this morphotype. For the “slender” morphotype, while the proportion of zoanthoxanthins was remarkably stable all along the year, we observed a significant increase in the concentration of parazoanthines in February (three-fold) and July (two-fold) while the concentration of ecdysteroids decreased in November, April and August (Figure 6). Parazoanthines as well as the other two families of compounds were always detected in this morphotype.

Focusing now on the temporal variation of the major compounds within each family for both morphotypes, several observations can be made. For both morphotypes, the slight increase in the production of zoanthoxanthins in December is mainly due to the increase of the major derivative paragracine (**8**) and to a lesser extent of parazoantoxanthin D (**6**) for the stocky morphotype. The increase in the ecdysteroids concentrations in both morphotypes during the periods of December to February and April to July is mainly due to the increase in the production of their major derivatives: 20-hydroxyecdysone (**1**) for the stocky morphotype and 20-hydroxyecdysone 3-acetate (**3**) for the slender morphotype. For the “slender” morphotype, the increase in parazoanthines during February and July is mainly due to the increase in the production of parazoanthine E (**13**) in relation to a slight decrease in the concentration of other analogues.

### Bioactivity of crude extracts and pure compounds

The natural bioactivity of crude organic extracts (Microtox®), expressed in EC_50_ values, ranges from 12.0 to 768 *µ*g/mL (Figure 7). The *P. axinellae* “slender” extracts (Average EC_50_ values: 35.4 *µ*g/mL) were significantly more bioactive than those of *P. axinellae* “stocky” (Average EC_50_ values: 372 *µ*g/mL). Among the three tested families of compounds, parazoanthines **9-16** showed a higher bioactivity (Average EC_50_ value: 8.51 *µ*g/mL) than ecdysteroids **1-4** (Average EC_50_ values: 24.9 *µ*g/mL) while zoanthoxanthins **5-8** did not show any activity at the initial concentration tested (1 mg/mL) against *Aliivibrio fisheri*.

## Discussion

**Figure 1:**
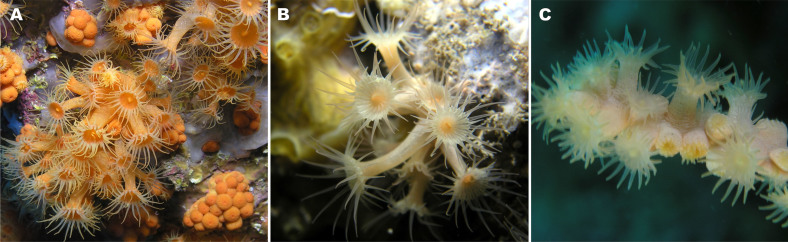
The two morphotypes of the Mediterranean zoanthid *Parazoanthus axinellae* studied here. A) “Stocky” morphotype growing in dense population, directly on the rocky substratum; B) “Slender” morphotype growing on the sponge *Cymbaxinella damicornis*; and C) *Savalia savaglia* growing on the gorgonian *Paramuricea clavata* (Pictures from T. Pérez).

Species identification in zoanthids is highly challenging, especially for species of the genus *Parazoanthus*. In the case of *P. axinellae*, a common zoanthid present in shallow waters of the Mediterranean, two morphotypes are easily distinguished: a bright yellow “slender” morphotype and a more orange “stocky” morphotype (Figure 1). The “slender” morphotype is the only one frequently found as epibiont of the sponges *Cymbaxinella damicornis* and *C. verrucosa*. Using molecular markers, Sinniger *et al.* extensively revised this group and showed that the genus *Parazoanthus* is polyphyletic and composed of three distinctive subclades^30^,^31^. However, most molecular markers used so far failed to absolutely discriminate at species level in zoanthids. This issue makes the distinction of closely-related species difficult^30^. The analysis performed in this study using six different molecular markers (mitochondrial 16S, 12S, COI 5′ region, COI 3′ region, 3′ region of the intron in COI and nuclear ITS-2) did not allow us to demonstrate any difference between “stocky” and “slender” morphotypes. These results are consistent with high conservatism of DNA sequence in zoanthids, providing evidence that there is no cryptic DNA variability between the studied morphotypes. In recent studies, metabolomics provided valuable information for the classification of groups of sponges^5^. We therefore applied an LC-MS metabolomic fingerprinting approach and used the diversity of the major secondary metabolites (in a defined retention time window) of *P. axinellae* to chemically distinguish its two morphotypes.

**Figure 2:**
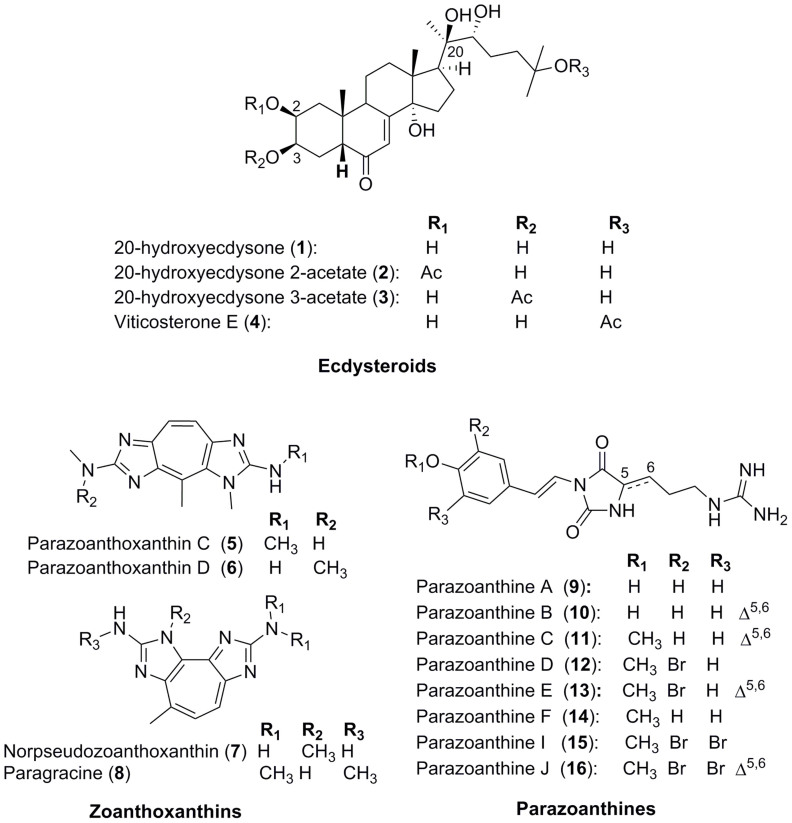
The three families of compounds isolated from *Parazoanthus axinellae*.

Relatively few chemical studies have been reported so far on *P. axinellae* although it is a widespread zoanthid, particularly in the Mediterranean Sea. In the 1970's, Cariello *et al.* provided the first results describing some components of the secondary metabolome of this species^20^,^21^,^22^. They isolated and characterized several zoanthoxanthin derivatives. For more than 30 years, no further chemical study were reported, but the chemical composition of *P. axinellae* was recently reinvestigated as this species is a keystone species of Mediterranean rocky beds. Recent advances in highly sensitive analytical tools were anticipated to allow the discovery of some additional minor metabolites. Indeed, the presence of a new family of hydantoin alkaloids named parazoanthines **9-16** was recently reported. In the present paper, we described the metabolome of this species in more details (Figure 2). We have identified some already known zoanthoxanthins, with minor differences observed between both morphotypes. Overall, zoanthoxanthin derivatives [paragracine (**8**) being always the major compound], are present at a higher concentration in the “stocky” morphotype. Parazoanthoxanthin C (**5**) in the “stocky” morphotype is replaced by norpseudozoanthoxanthin (**7**) in the “slender” one while parazoanthoxanthin D (**6**) is present in both morphotypes (Figures 6). Different compositions in these already described pigments can explain the differences in colours between the bright yellow “slender” morphotype and the orange “stocky” one. For the first time the presence of ecdysteroids **1-4** is reported in this species and, in this case also, at higher concentrations for the “stocky” morphotype (Figures 6 and 7). The production of ecdysteroids by *P. axinellae* is not surprising, as several members of this chemical family have already been reported from other species of the genus *Parazoanthus* and mostly from other zoanthids^32^. Because ecdysteroids have already been described from insects and crustaceans as hormones^33^,^34^, they may have an analogous role in this zoanthid. The composition in ecdysteroids also differs between morphotypes. While 20-hydroxyecdysone (**1**) appears as the major compound in the “stocky” morphotype, the acetylated forms of 20-hydroxyecdysone **2** and **3** are the major ecdysteroids in the “slender” morphotype. On the contrary, the occurrence of parazoanthines **9**-**16** is intriguing since: i) they are the major secondary metabolites of the crude extracts of the “slender” morphotype, and they are never detected in the “stocky” morphotype; ii) as parazoanthines and zoanthoxanthins have similar polarities, the earlier chemical studies which reported the presence of zoanthoxanthins should have also identified parazoanthines if present (Figures 6)^20^,^21^,^22^. The extraction conditions cannot explain the absence of these metabolites in the earlier studies since ours were very similar. We therefore believe that the samples studied in the 1970s were certainly specimens of the “stocky” morphotype.

Wherever we sampled across the western basin of the Mediterranean Sea, metabolomic profiles of two different morphotypes showed constant chemical divergence with the presence of parazoanthines restricted to the “slender” morphotype (Figures 4 and 5). As a consequence, the occurrence of parazoanthines cannot be related to the sampling site. Sinniger *et al.* proposed that the substratum where zoanthids grow (especially when biological) could be a key factor in the evolutionary history of some species^30^,^31^. In our study, all the “stocky” *P. axinellae* samples were collected on rocky substrates, mostly of biogenic origin (coralligenous bioconstructions). About half of our “slender” *P. axinellae* samples were collected from the rocky substrate as well and the remaining samples were found in epibiosis on the sponge *Cymbaxinella damicornis*. From a chemical point of view, it clearly appears that the substrate from which adult individuals were collected does not induce any chemical difference between specimens. The “slender” samples growing on the rocks did not show a chemical fingerprint distinct from the “slender” samples that were collected on sponges. However, it should be noted that *P. axinellae* is known to expand and colonize rocky substrates mostly asexually with very frequent fission events leading to fragmentation of colonies and “dropping” polyps^35^,^36^. Observations of such events mostly come from “slender” colonies. It is therefore possible that, even when found on biogenic “rock”, the “slender” morphotype may actually derive from asexual reproduction of individuals originally settled on *Cymbaxinella* sponges. However, it should be noted that the reproduction, larval settlement and life cycle are virtually unknown in *P. axinellae*, so this hypothesis remains to be tested

The temporal study over one year confirmed the presence of parazoanthines **9-16** in the “slender” morphotype and their absence in the “stocky” morphotype across all time points (Figure 6). A clear increase in the production of parazoanthines was observed in February, which is mainly inferred to the high production of the oxidized parazoanthine E (**13**) while its associated reduced form, parazoanthine D (**12**), is the major component along the rest of the year. An oxidation step, acting certainly through the activation of an oxidase, seems to be triggered at this moment of the year. Furthermore, temporal series enabled the assessment of seasonal variation in the production of the less concentrated zoanthoxanthins and ecdysteroids. A slight increase in the production of the pigments zoanthoxanthins and the ecdysteroidal hormones in winter may possibly be related to the reproductive cycle but no clear change in their relative composition was observed along the year^37^. The temporal variability of LC-MS fingerprints within the “slender” morphotype was essentially related to the occurrence of parazoanthine E (**13**) and some other minor unknown compounds (peak height < 10^6^ on the recorded LC-MS profile). Also, all HPLC-MS chromatograms exhibiting a small quantity of unknown alkaloids (peak height < 10^6^) corresponded to *P. axinellae* “slender” collected as epibionts of *Cymbaxinella* sponges (HPLC-MS chromatograms not shown). The chemical diversity of these sponges is rather well characterized and a large panel of pyrrole 2-aminoimidazoles derivatives have been characterized from both species^38^. These unknown alkaloids with different *m/z* can be ascertained not to belong to the host sponge through sample contamination. The presence of these minor compounds in the “slender” morphotype may then be the result of a strong interaction between the sponges and the zoanthids. Even if the relationships between *P. axinellae* and *Cymbaxinella* sponges has long been known and reported, no comprehensive study has ever been undertaken. An in-depth chemical ecology study would be necessary to better understand the putative molecular/biochemical nature of the interactions between *P. axinellae* and *Cymbaxinella* sponges.

The bioactivity of *P. axinellae* “slender” crude extract, assessed using the standardized Microtox® bioassay, was almost 10 times higher when compared to *P. axinellae* “stocky” (Figure 7). Parazoanthines appeared to be the most bioactive family of compounds, followed by ecdysteroids and zoanthoxanthins, which did not exhibit any bioactivity for the initial concentration tested. The difference in bioactivity of both morphotypes is thus due to the occurrence of parazoanthines. It would be of great interest to explore the possible pathways by which the sponge and the zoanthid might exchange chemical compounds. We could also assume that the epibiosis triggered the expression of a biosynthetic gene leading to parazoanthines. The production of these compounds might help the zoanthid to overcome the sponge antifouling activities through an imbalance in the defensive arsenal of the bacteria/sponge holobionts. Moreover, the tissues of the sponges are clearly affected by the presence of this zoanthid. Thus, we can propose the two following hypotheses: i) the gene leading to parazoanthines is vertically transferred only in the “slender” morphotype (that could eventually lead to two distinct species) or; ii) such a gene exists in both morphotypes, but is silent when *P. axinellae* does not settle as an epibiont of the sponge. Important additional studies must be performed to explore these hypotheses. Molecular biology could be used to target the genes of biosynthetic pathways leading to parazoanthines. In our previous paper we proposed two metabolic pathways for parazoanthines^23^. Key biochemical transformations involved in the biosynthesis like the formation of the peptidic bonds or the cyclization by already described hydantoinases could therefore represent the first targets^39^,^40^.

In this study we demonstrated a strong chemical divergence between two morphotypes of the zoanthid *P. axinellae*. LC-MS metabolomic profiling showed that the presence of recently described parazoanthine alkaloids is a characteristic trait of the “slender” morphotype. These results once more raise the question about the specific status of *P. axinellae* morphotypes, which differ not only by their morphological traits (colour, shape) but now also by their secondary metabolome. The search for congruence between multiple and complementary sources of taxonomical data is the basis of an approach called integrative taxonomy^41^,^42^. A non-targeted metabolomic approach was already applied for the classification of Homoscleromorpha, but in the case of *P. axinellae* we were able to describe the structure of most of the metabolites present in our metabolomic window. Remarkably, this “so-called” targeted metabolomic approach allowed us to distinguish two morphotypes associated to two chemotypes within the same species, with only one morphotype producing an entire family of metabolites known as parazoanthines. The present work is thus a first significant step towards resolving a putative species complex, with parazoanthines used as specific chemotaxomic markers, but complementary datasets (*e.g.* molecular evidences, reproduction traits, chemical interactions, biosynthetic pathways) are still needed to definitely revise the status of the Mediterranean *P. axinellae* morphotypes.

## Methods

### Biological Material

*Parazoanthus axinellae* (Schmidt, 1862) is a macrocnemic zoanthid of the family Parazoanthidae (Cnidaria, Hexacorallia, Macrocnemina, Parazoanthidae). Two morphotypes of *P. axinellae* have been targeted in this study. They can be easily identified in the Mediterranean Sea by their external morphology. Mostly encountered on the rocky substrate, “slender” morphotype (Figure 1B) is also often found as an epibiont on the demosponges *Cymbaxinella damicornis* and *C. verrucosa.* Its polyps are rather yellow and thin, the tentacles elongate. The bright orange *P. axinellae* “stocky” morphotype (Figure 1A) is mainly found on the primary, generally bioconstructed, rocky substrate, where it can form dense populations covering up to 100% of the available surface. It is only rarely observed growing on demosponges (mostly *Petrosia ficiformis*) but never on *Cymbaxinella damicornis* and *C. verrucosa*. *Savalia savaglia* (Bertoloni, 1819), a third species of the family Parazoanthidae has been used as an outgroup for classification purposes (Figure 1C). It is also a macrocnemic zoanthid living on gorgonian axes, found in the Mediterranean and North Eastern Atlantic.

### Sampling

**Figure 3:**
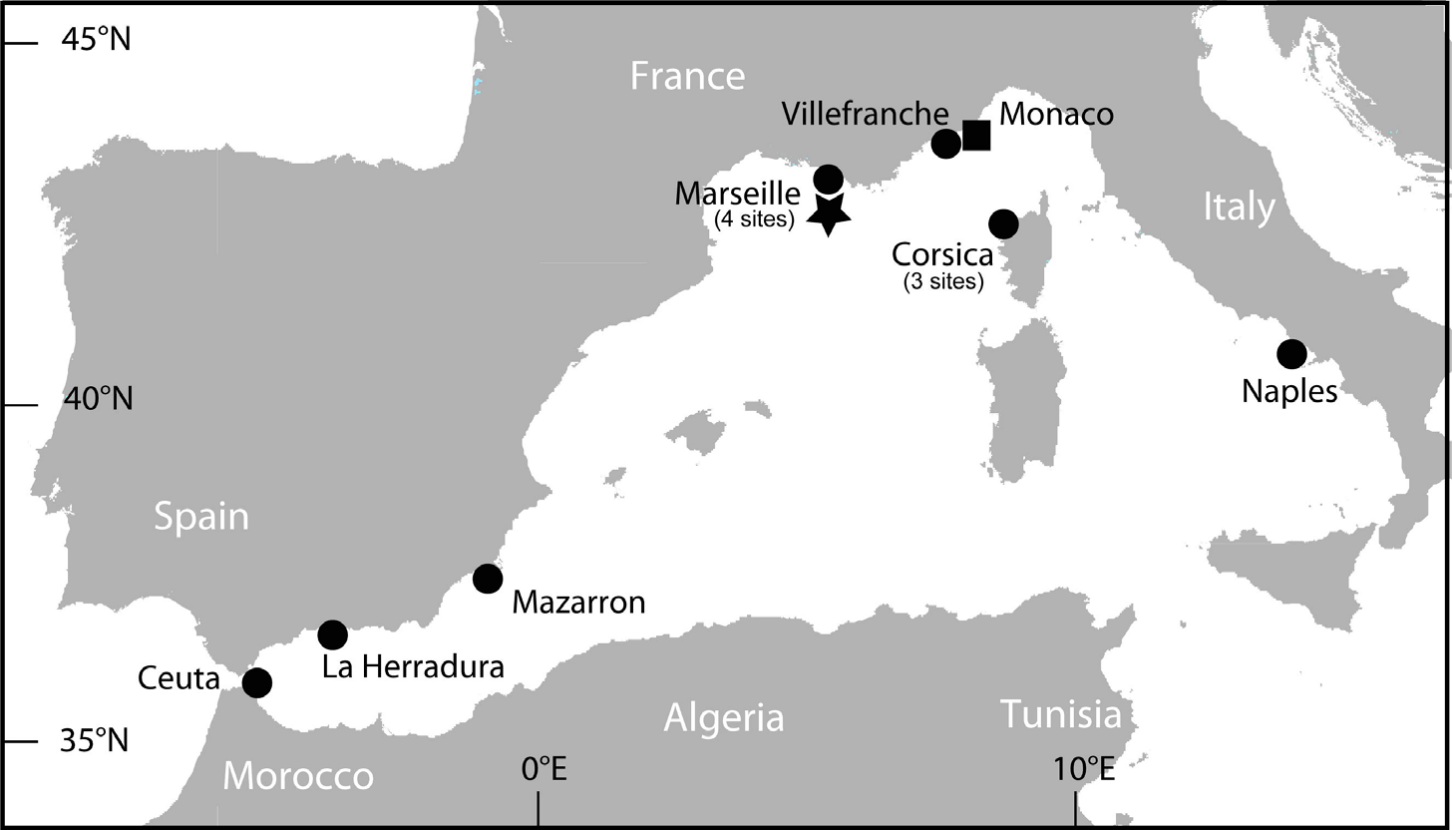
Map of the Western Mediterranean with *Parazoanthus axinellae* sampling locations indicated as black circles (see details in Table 1). Black square represents the site (Mo1) where a 1-yr time series was conducted. Black star represents the sampling location for the outgroup *Savalia savaglia* (Marseille). This map was obtained from http://d-maps.com/carte.php?num_car=3122&lang=fr (Date of access: 16 Apr. 2013) and modified under Adobe Illustrator.

**Figure 4:**
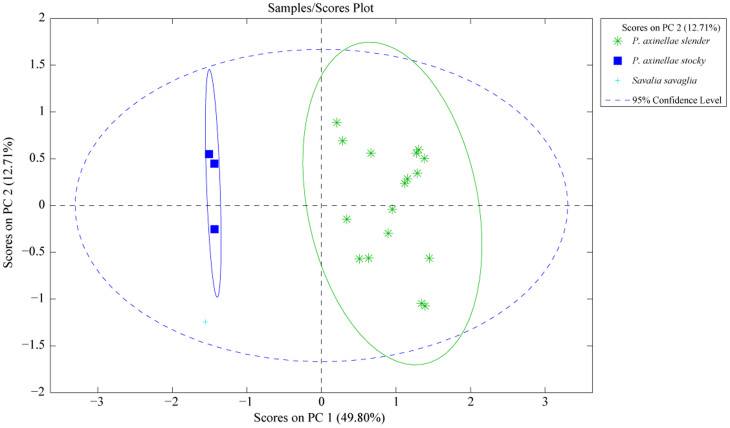
Principal Component Analysis (PCA) of metabolomic profiles of both *P. axinellae* morphotypes (17 “slender” and 9 “stocky” morphotypes) according to the location (score plots).

**Figure 5:**
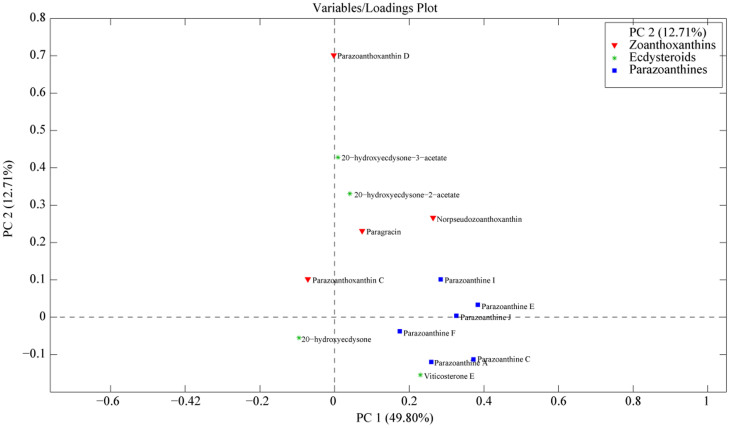
Principal Component Analysis (PCA) of metabolomic profiles of both *P. axinellae* morphotypes (“slender” and “stocky”) according to the location (loading plots).

**Figure 6:**
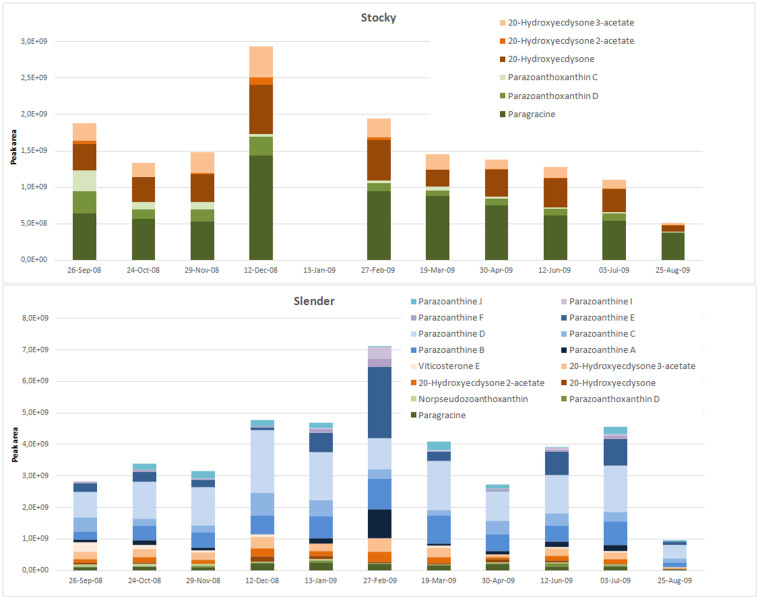
Temporal variation (one annual cycle) of three target metabolite families for the “stocky” and “slender” morphotypes. Metabolite expression level is displayed as peak area in the Base Peak Chromatogram (no data are available for the stocky morphotype on the 13^th^ January 2009).

**Figure 7:**
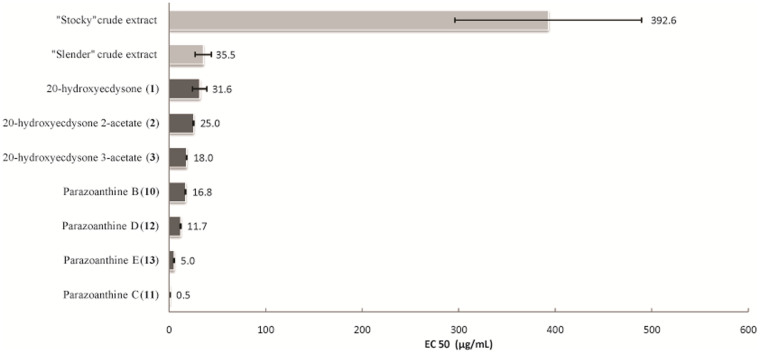
Bioactivity assay (Microtox®) performed on extracts and pure compounds from *P. axinellae*.

All samples were collected by SCUBA diving. To study the intra-specific variability of the *P. axinellae* metabolome over time, samples of both morphotypes were collected monthly and randomly from September 2008 to August 2009 at a specific site off the Monaco coast where they were found in close vicinity and similar environments (Station Mo1, “Roches Saint Nicolas”, vertical cliff at 19 m depth) (Figure 3). At this station, 21 samples of *P. axinellae* (11 of *P. axinellae* “slender” and 10 of *P. axinellae* “stocky”) were collected in triplicate. For the spatial variability study, 26 samples of *P. axinellae* (17 samples of *P. axinellae* “slender” and 9 of *P. axinellae* “stocky”), from 13 different sites in the Mediterranean (Table 1, Figure 3), were collected approximately at the same period of the year between May and June. For specimens found on sponges, the separation was performed very carefully in order to avoid contamination from the host tissues.

### DNA Analysis

DNA sequence variability at several loci was explored with stocky and slender specimens from Marseille. Samples of *S. savaglia* were obtained in one site near Marseille in July 2007 and June 2008. Samples for DNA study were stored in 95% ethanol upon collection. DNA was extracted, PCR amplified and sequenced for DNA fragments from the mitochondrial (12S, 16S, COI) and nuclear (ITS-2) genomes. PCR primers and conditions are found in previous reports^30^,^31^. In addition to these known markers, the intergenic region preceding the HEG gene located in the intron within COI as well as the 3′ end of COI were sequenced using the primers COI2a and BB5moR^43^. Sequences were aligned and edited with BioEdit 7.1.9^44^. All new and updated sequences are in GenBank under accession numbers: AB247355, AY995912, AY995935, EF672659, EF687826, EU363364, EU591571 and LN606759 – LN606763.

### Metabolomic analyses

#### Chemicals

Methanol, dichloromethane (both Chromasolv®, gradient grade) and formic acid (Fluka, puriss. P.a. ~98%), were provided by Sigma-Aldrich. Ultrapure water was prepared using a Milli-Q water system (Millipore Ltd.).

#### Sample preparation for metabolomic studies

Each sample was immediately frozen at −20°C after collection. They were then freeze-dried and ground to obtain a homogenous powder. 0.2 g of each sample powder was extracted three times in a row for 2 minutes with 2 mL of CH_2_Cl_2_/MeOH 1:1 (v/v) in an ultrasonic bath at room temperature (20°C). The filtrates of each extraction were combined. 2 mL of the global filtrate were used for HPLC-UV-MS analyses and 1 mL was used for a bioactivity assessment with the Microtox® assay.

#### Chromatographic analyses

On-line HPLC-UV-MS analysis was performed using a Waters 2696 (Alliance) system equipped with an autosampler and a Waters 2487 dual absorbance wavelength detector, linked to an ion trap mass spectrometer fitted with an electrospray ionization interface (Bruker Esquire 3000 Plus). Mass spectra were recorded in the positive mode. HPLC separation was achieved on an analytical Phenomenex Gemini C_6_-Phenyl column (250 × 3 mm, 5 *µ*m) using a linear elution gradient of H_2_O/MeOH/formic acid from 90:10:0.1 (v/v/v, isocratic from 0 to 5 min) to 0:100:0.1 (v/v/v, isocratic from 35 to 45 min, flow rate 0.5 mL/min) in 30 min. The injected volume was set at 10 *µ*L and one of the selected wavelength was set at 254 nm because all major compounds detected in an additional HPLC-DAD-ELSD experiment absorbed at 254 nm. The mass spectrometer analyzer parameters were set as follows: nebulizer sheath gas, N_2_ (50 psi); dry gas, N_2_ (9 L/min); capillary temperature, 350°C; capillary voltage, 4500 V; skimmer voltage, +40 V; lens 1 voltage, -5 V; lens 2 voltage, −60 V; collision gas, He; ion trap target, 30,000; the ion charge control (ICC) was on. Target mass was set to *m/z* 500 and full scan data were collected between *m/z* 50 to 1200.

#### Purification and Structure Identification of the Major Metabolites

The chromatogram obtained between 10 and 30 min of retention time was considered as our *metabolomic window* and the major compounds of this zone were thoroughly identified. HPLC purification were carried out on a Waters 600 system equipped with a Waters 996 photodiode array detector coupled with a Sedex 55 ELSD (SEDERE, France), and a Waters 717 plus autosampler. The compounds isolation was achieved by HPLC on a semi-preparative RP-column (Phenomenex, Luna C_18_, 250 × 10 mm, 5 *μ*m) with a linear elution gradient of H_2_O/MeOH/TFA from 30:70:0.1 to 28:72:0.1 (v/v/v) in 30 min at a flow rate of 3.0 mL/min. NMR experiments were performed on a Bruker Avance 500 MHz spectrometer.

#### Data Analysis

LC-MS raw data were processed by Data Analysis software (Bruker). Base Peak Chromatograms (BPC) were exported as line spectra and converted to netCDF file format to process unit mass resolution data in centroid mode with MZmine 1.96 Toolbox. MZmine peak detection was achieved by centroid mass detection using a parameter of “noise level” set to identify the representative data point of each peak in spectrum domain. This value sets the minimum of intensity for a centroid data point to be considered as part of a peak level (noise level = 10^5^). The highest intensity chromatogram builder was used to eliminate the noise and define each peak by “min time span” (20 s), “min height” (10^6^) and “*m/z* tolerance” (0.1). The peak deconvolution was performed using “baseline cut off” respecting the following parameters: “min peak height” 10^6^, “min peak duration” 20 s, “baseline level” 10^5^. As some secondary metabolites contain bromine, the “isotopic peak grouper” was also performed [*m/z* 2,000, “RT (Retention Time) tolerance” 20 s, “monotonic shape” on, “max charge” 1, “representative isotope” lowest *m/z*]. Alignment was carried out using “absolute RT tolerance” of 1 minute, and ”*m/*z tolerance” of 0.3. Each known metabolite was then identified on the basis of its *m/z* and RT and the data matrix was filtered to keep only the identified peaks in a RT range of 10–30 min. The filtered data matrix was transformed into discrete binary (presence/absence) characters. It was then exported with each compound as a variable, and their presence or absence (1 or 0) as values. To monitor the variation of targeted metabolites over time, the quantity of each metabolite was expressed as a peak area per sample dry mass (200 mg).

#### Statistical Analyses

Non parametric Kruskal-Wallis tests (KW) were applied to assess the temporal variation of targeted metabolites in each morphotype. Principal Component Analyses (PCA) were applied to explore and identify patterns in chemical diversity across *P. axinellae* samples. Hierarchical Cluster Analysis (HCA) was performed to classify samples on the basis of their metabolomic profile similarities, and to determine relationships between different morphotypes of *P. axinellae* and *S. savaglia*. Multivariate data analyses (PCA and HCA) were performed with Primer-E 6 Ltd. software (Plymouth Marine Laboratory, 2005).

### Bioactivity assay

The standardised Microtox® bioassay (Microbics) was used to assess the ecological bioactivity of pure target compounds from the three families (ecdysteroids, zoanthoxanthins and parazoanthines), and crude extracts (CH_2_Cl_2_/MeOH, 1:1, v/v) of each *P. axinellae* morphotype (3 samples per morph)^45^. Pure compounds or crude extracts were dissolved in artificial seawater and up to 1% acetone was added to improve their dissolution. Solutions were tested in four diluted concentrations. A dilution factor of 2 was applied between each following tested concentration. The initial concentration was set to 1000 *µ*g/mL for crude extracts and between 200 and 25 *µ*g/mL for pure compounds. The bioactivity was quantified by measuring the direct effect on the metabolism of the bioluminescent bacterium *Aliivibrio fischeri* indicated by a decrease in light emitted and expressed as an EC_50_ value (Microtox assay®).

## Author Contributions

O.P.T., T.P., P.C., G.G.-J. and N.C. designed the experiment. G.G.-J., N.C. and F.S. performed the experiments. G.G.-J., N.C., F.S., G.C. and J.I. analyzed the data. N.C., O.P.T., T.P. and P.C. wrote the paper. All authors discussed the results and commented on the manuscript at all stages.

## Supplementary Material

Supplementary Information SI

## Figures and Tables

**Table 1:** Geographical information on sampling sites of *P. axinellae* across the Mediterranean

Sampling site	Site ID	Latitude N	Longitude
*Marseille*			
Riou, Impérial de Terre	Ma1	43°10′23.797″	5°23′34.436″E
Plane, Grotte Pérès	Ma2	43°11′15.719″	5°23′17.159″E
Jarre, Grottes Mysid & Arc-en-Ciel	Ma3	43°11′51.968″	5°21′53.813″E
Grotte du Chinois, Niolon	Ma4	43°20′16.380″	5°15′25.440″E
*Villefranche-sur-Mer*			
Grotte du Lido	Vf1	43°41′31.487″	7°19′12.186″E
*Monaco*			
Roches St-Nicolas	Mo1	43°44′10.450″'	7°25′59.560″E
*Corsica*			
Pointe de la Revellata	Co1	42°35′5.265″	8°43′39.047″E
Calvi, La Bibliothèque	Co2	42°34′6.586″	8°44′17.862″E
Golfe de Porto, Calanque de Piana	Co3	42°15′ 4.270″	8°36′52.553″E
*Naples*			
Nisida	Na1	40°47′38.378″	14°9′35.428″E
*Costa blanca*			
Mazarron	Cb1	37°31′29.760″	1°7′24.720″W
La Herradura, Punta Cerro Gordo	Cb2	36°43′45.780″	3°45′55.805″W
*Ceuta*			
Punta Almina	Ce1	35°54′51.005″	5°16′42.907″W

## Notes

Hierarchical Cluster Analysis for geographical variation, LC-UV-MS profiles of both morphotypes.
