# Supplementary material for: Metabolomic profiling reveals deep chemical divergence between two morphotypes of the zoanthid Parazoanthus axinellae
Source: Sci Rep. 2015 Feb 6;5:8282. doi: 10.1038/srep08282 (PMC4319174; doi:10.1038/srep08282)
Supplement: Supplementary Information — SI [file srep08282-s1.pdf]

Supporting Information for

Metabolomic profiling reveals deep chemical divergence between two morphotypes of the zoanthid *Parazoanthus axinellae*

Nadja Cachet, Grégory Genta-Jouve, Julijana Ivanisevic, Pierre Chevaldonné, Frédéric Sinniger, Gérald Culioli, Thierry Pérez & Olivier P. Thomas

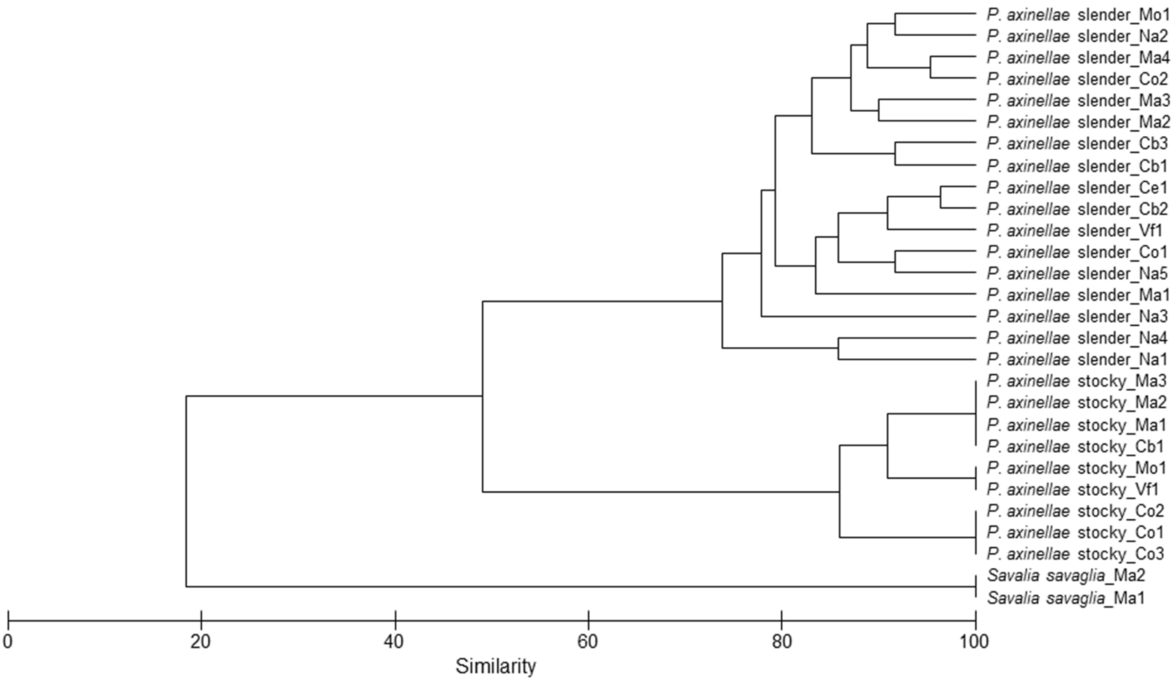

Hierarchical Cluster Analysis of the metabolomic profiles of borth morphotypes of *P. axinellae*.

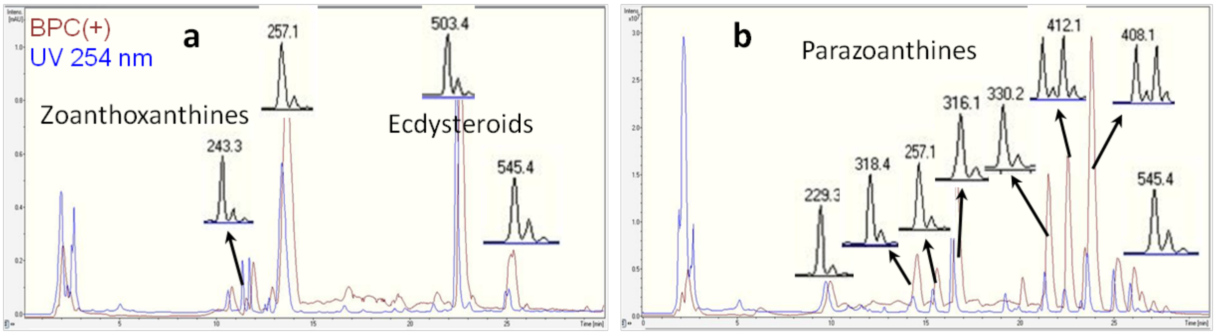

Example of HPLC-UV-MS profiles for a) the stocky morphotype and b) the slender morphotype
